# Eukaryotic microalgae-bacteria synthetic consortia boost crop productivity and drought tolerance in bread wheat (*Triticum aestivum*)

**DOI:** 10.3389/fpls.2025.1726084

**Published:** 2026-01-07

**Authors:** Celeste Molina-Favero, Lara Sanchez Rizza, Angie Melissa Gonzalez Olano, Guillermo Maroniche, Mauro Polizzi, Eduardo de Gerónimo, Cecilia Creus, Leonardo Curatti, Luciana Anabella Pagnussat

**Affiliations:** 1 Instituto de Innovación para la Producción Agropecuaria y el Desarrollo Sostenible, Instituto Nacional de Tecnología Agropecuaria-Consejo Nacional de Investigaciones Científicas y Técnicas (IPADS, INTA-CONICET), Balcarce, Argentina; 2 Instituto de Investigaciones en Biodiversidad y Biotecnología (INBIOTEC-CONICET), Fundación para Investigaciones Biológicas Aplicadas (FIBA), Mar del Plata, Argentina; 3 Consejo Nacional de Investigaciones Científicas Científica y Técnicas (CONICET), Mar del Plata, Buenos Aires, Argentina; 4 Laboratorio de Bioquímica Vegetal y Microbiana, Facultad de Ciencias Agrarias, Universidad Nacional de Mar del Plata, Balcarce, Buenos Aires, Argentina

**Keywords:** microalgae, wheat, plant growth promoting rhizobacteria, drought, rhizosphere

## Abstract

Wheat provides the main source of nourishment for more than 40% of the global population, making it an essential crop. The challenge of overseeing crop management to guarantee water efficiency has been enhanced by the increase in rainfall unpredictability caused by climate change. Plant-growth-promoting bacteria (PGPBs) are beneficial microorganisms capable of improving crop yield and adaptability to environmental stresses. Single-celled eukaryotic algae, on the other hand, are comparatively understudied organisms that exhibit plant-biostimulant properties. Our research demonstrates that co-inoculation of *Azospirillum argentinensis* Az39 with the microalgae *Scenedesmus obliquus* C1S increases bacterial root colonization and the sole inoculation with microalgae improves germination and post-germinative growth under drought conditions. Field trials conducted on 2022 and 2024, under the influence of environmental drought conditions, revealed a 36% boost in grain yield and a 26.2% improvement in crop water productivity resulting from inoculation with microalgae-PGPB consortia. Moreover, under induced drought conditions, seedlings inoculated with microalgae showed a 50% increase in root dry weight. Notably, our results also reveal that inoculation efficiency was affected by tillage methods. The findings presented herein disclose a promising potential for the development of a novel eukaryotic microalgae-PGPB synthetic consortia inoculant that enhances root colonization by PGPBs and improves wheat crop water productivity in the field.

## 1 Introduction

Wheat is a major worldwide crop, representing over 20% of the calories and proteins harvested worldwide (Shiferaw et al., 2013) and providing as the primary food supply for over 40% of the global population (Zhang et al., 2022). Contemporary management approaches predominantly depend on the utilization of external inputs, including insecticides for pest and disease management, mineral fertilizers to enhance plant nutrition and biomass, and sometimes irrigation to mitigate water stress circumstances (You et al., 2022). The yield of rain-fed crops is linked to the precipitation they receive throughout their growth cycle (Andrade and Satorre, 2015). Thus, the careful monitoring of crop management to ensure water efficiency and alleviation of the negative effects of water scarcity is crucial for sustainable crop production. This challenge is amplified by the anticipated increase in rainfall variability due to climate change, which, in the case of wheat bread, can decrease production up to 60% (Aramburu Merlos et al., 2015). In the southern hemisphere, the potential grain surplus due to appropriate irrigation is highly variable due to the influence of the El Niño-Southern Oscillation phenomenon (ENSO). ´El Niño´ phase is reflected as an increase in spring/summer rainfalls and higher summer crop yields, while the opposite occurs with “La Niña” events, which typically result in dry years. In this context, farmers face significant challenges due to environmental unpredictability, since different seasons require different management strategies based on rainfall patterns and timing (Edreira et al., 2018). Conservation tillage has gained popularity in recent years due to its efficacy in mitigating soil degradation and enhancing soil water retention (Hashimi et al., 2023). No-tillage (NT) systems might have potential benefits over conventional tillage (CT) systems under specific management situations. These advantages encompass a diminished number of machine runs over the field, enhanced aggregate stability, the protective influence of agricultural residues remaining on the soil (Sithole et al., 2019), enhancing soil water retention, and a greater prevalence of biopores (Blanco-Canqui and Ruis, 2018; Hashimi et al., 2023). However, when subjected to NT management, certain soils might suffer adverse impacts, including heightened bulk density on the 0–20 cm layer of the soils (Díaz-Zorita et al., 2002; Martínez et al., 2008), and diminished oxygen diffusion rates (Khan and Science, 1996).

Plant-growth-promoting bacteria (PGPBs) are a group of rhizospheric beneficial bacteria that have the potential to enhance crop productivity and acclimation to abiotic stress through multiple mechanisms (Bhattacharyya and Jha, 2012; Cassán et al., 2014). *Azospirillum argentinensis* has a direct influence on the plant, via the synthesis of several phytohormones, including indole acetic acid (Spaepen et al., 2007), abscisic acid (Wu et al., 2025), gibberellins (Cohen et al., 2009), salicylic acid (Sahoo et al., 2014), cytokinin (Zaheer et al., 2022) and nitric oxide (NO) (Molina-Favero et al., 2008). On the other hand, fluorescent pseudomonads, major inhabitants of the rhizosphere, have both direct and indirect favorable impacts on plant development (Choudhary et al., 2009) Pseudomonas strains synthesize a diverse array of chemicals exhibiting antibacterial properties, with 2,4-diacetylphloroglucinol (DAPG) being among the most extensively researched (Weller et al., 2007). These PGPBs are presently marketed as inoculants, and strain compatibility is an absolute requirement to achieve increased plant growth promotion by co-inoculation of several PGPBs (Pagnussat et al., 2016; Díaz et al., 2023). A critical aspect of this strategy is that PGPBs inoculated onto seeds must survive on the dry or semi-dry surface tissues of the seed until appropriate conditions emerge for root colonization. Consequently, inoculants often require adjuvants or seed-coating treatments to enhance their viability (Rocha et al., 2019).

Although much less studied than bacteria, single-celled eukaryotic algae (commonly known as microalgae) also exhibit plant biostimulant properties. Microalgae are widespread in soils, contributing to their organic carbon content, structure, and moisture. In addition to releasing plant hormones and other growth-stimulating substances, some microalgae produce a complex matrix of EPS. These EPS are believed to shield co-inhabiting bacteria from desiccation and UV radiation, in a microenvironment that provides them with essential nutrients and hormones for their survival and growth, and drive complex mutualistic interactions within microalgae-bacteria consortia (Perera et al., 2022). In this sense, when co-cultured, auxins produced by *Azospirillum baldaniorum* Sp245 mitigate the oxidative stress of the microalgae *Scenedesmus obliquus* C1S, under both saline stress (Pagnussat et al., 2023) and N deficiency (Pagnussat et al., 2020), thereby promoting the growth of the microalgae. Additionally*, A. baldaniorum* and *S. obliquus* engage in mixed biofilms, a property which could contribute to the higher rates of bacterial survival observed in co-culture, particularly under salinity stress (Pagnussat et al., 2023).

Field inoculation of crops with beneficial microorganisms is a sustainable approach to improve crop productivity and stress resilience. Nonetheless, PGPB performance may fluctuate due to environmental variables, microbial competition, and agronomical practices (Bulgarelli et al., 2013). In recent studies, tillage practice was identified as the primary factor influencing the microbiome in the rhizosphere (Behr et al., 2024). Moreover, on wheat soils, tillage methods have a significant interactive effect with the water regime on root-associated bacterial and fungal populations (Romano et al., 2023), underscoring the enduring legacy of tillage practices likely attributable to variations in physical soil properties and chemical composition. This highlights the necessity for additional research to improve inoculation techniques to provide reliable agricultural benefits.

Considering the biostimulant properties of microalgal exudates (La Bella et al., 2022), we hypothesize that microalgae can offer emergent properties to multispecies PGPB inoculants. In this work, we explored whether microalgae incorporated into bacterial inoculants can (i) favor the survival of bacteria in the rhizosphere, and (ii) improve wheat water productivity under field conditions and different tillage managements. Our results establish, for the first time, the potential of microalgae-bacteria communities as bioinputs for crops in the field, an area that still remains unexplored and holds significant implications for developing novel multi-species inoculants.

## 2 Materials and methods

### 2.1 Microorganisms and growth conditions

*Scenedesmus obliquus* C1S (Do Nascimento et al., 2012) and *Azospirillum argentinense* Az39 and *Pseudomonas* sp. LSR1 were used as study microorganisms. *S. obliquus* C1S was routinely cultured in BG11 medium containing 10 mM NaNO_3_ as a nitrogen source and 0.42 g x L^-1^ NaHCO_3_ to buffer CO_2_ supplementation. Recombinant fluorescent derivatives of *A. argentinense* Az39 and *Pseudomonas* LSR1, Az39-dsRED (Puente et al., 2021) and LSR1-eGFP (Maroniche et al., 2018) were also used when required. All experiments- whether single, double, and triple treatments- were initiated with 10^6^ cells of *S. obliquus* C1S, counted under a light microscope (Leica DM500, Germany) on a Newbauer chamber, 10^6^ cells of *A. argentinensis* Az39, and/or *Pseudomonas* sp. LSR1 (estimated by optical density at 600 nm, OD_600_) per seed. Starter single-species cultures of Az39 and LSR1 were cultured in Luria-Bertani medium (LB) without salt at 30°C with orbital shaking (150 rpm) for 18 h. Tetracycline at 25 μg mL^−1^ was included in the medium for culture of the recombinant fluorescent strains.

### 2.2 Seed inoculation and root colonization

Wheat seeds (*Triticum aestivum* (L.) cv. Macro-Seed Instituto Nacional de Tecnología Agropecuaria 221 (MS INTA 221, long-cycle cultivar) and cv. MS INTA 819 (short-cycle cultivar) were used for the 2022 campaign and growing chamber trials, and the 2024 campaign, respectively. Seeds were superficially sterilized (10 min with 50% sodium hypochlorite) and inoculated with a suspension of microalgae alone, bacteria alone, bacteria consortium, or with a triple consortium (microalgae plus both bacterial strains). Fluorescent bacterial variants were used for root colonization analysis. Previous investigations have found no effect of the fluorescent protein tag on bacterial fitness or colonization capacity (Ramirez-Mata et al., 2018; Puente et al., 2021; Fernández et al., 2022). Suspensions with microorganisms were applied to the seeds at a final inoculation volume of 5 μL per seed, and stored on paper envelopes at 25°C for 24 h before sowing.

Root colonization was evaluated 3 days after germination on agar-water plates. Roots were cleaned and crushed in a mortar, and bacterial colony-forming units (CFU) were counted. g^-1^ were evaluated using the microdrop technique as previously described (Herigstad et al., 2001). Roots colonized by fluorescent bacteria (Az39-dsRED and LSR1-eGFP were also directly observed with a Nikon C1 confocal laser microscope. LSR1-egfp and Az39-dsred bacteria were excited/detected at 488/550 nm and 543/650 nm, respectively. Images were analyzed using Nikon EZ-C1 Free viewer software.

### 2.3 Microalgae localization and viability

*Scenedesmus. obliquus* C1S localization and viability were examined in radicle-protruding seeds at one day post-imbibition (1 dpi). Transversal sections of the seed trichomes region, each one- one-millimeter thick, were stained with SYTOX Green at a final concentration of 1 µM for 30 min in the dark at 4°C. They were subsequently analyzed using light and fluorescence microscopy with a Nikon E600 microscope, equipped with a B-2A cube that includes 450–490 nm excitation and 500–515 nm emission filters, utilizing a 40.0xA/1.25/0.17 oil-immersion Nikon lens. Images were captured using an Olympus DP72 digital camera and Cellsens Entry imaging software.

### 2.4 Analysis of microalgal phytohormone profile by ultra-high-performance liquid chromatography coupled to tandem mass spectrometry

Microalgal cells were resuspended in 1.5 mL 1 M NaCl, sonicated at a 50% power-output (Vibra-Cell, model VCX-130, Sonics Inc.) for three cycles of 1 min each (10 sec on, 1 sec off), immediately frozen with liquid nitrogen, and lyophilized. Jasmonic acid (JA), abscisic acid (ABA), salicylic acid (SA), indole-3-acetic acid (IAA), cis-zeatin (cZ), cis-zeatin riboside (cZR), trans-zeatin (tZ), trans-zeatin riboside (tZR), and gibberellic acid (GA_3_) were extracted using 100 mg of lyophilized microalgae. The samples were processed according to (Giannarelli et al., 2010), and the resulting extracts were diluted 10-fold with ultrapure water, filtered through a 0.22 µm nylon filter, and analyzed. Phytohormone concentrations were determined by UHPLC (UHPLC ACQUITY I-Class UPLCTM) coupled to tandem mass spectrometry (XEVO TQ-XS) equipped with an ACQUITY UPLC HSS C18 Column (1.8 μm, 100 x 2.1 mm) (Waters). The mobile phases were water: methanol 95:5 (phase A) and methanol (phase B), both modified with ammonium acetate 0.1 mM and formic acid 0.01% v/v. The flow rate was set at 0.3 mL.min^−1^ and the column temperature was 45°C. The chromatographic separation was performed with the following gradient elution conditions: B was 10% (v/v) in 0−0.5 min, linearly increased to 90% (v/v) in 0.5−11 min; held at 90% for 11−12.5 min, and returned to the initial condition in 1.5 min.

An auto-sampler was used to inject 10 μL of the samples. The XEVO TQ-XS tandem quadrupole mass spectrometer was operated in positive and negative mode with the electrospray-ionization (ESI) source. The operating parameters were optimized under the following conditions: capillary voltage, 3 kV, ion source temperature 150°C, desolvation temperature 500°C, cone gas flow 150 L. h^−1^, desolvation gas flow 800 L h−1 (both gases were nitrogen obtained from a nitrogen generator) and collision gas flow 0.15 mL min−1 (argon gas 99.995% with a pressure of 4.04×10−3 mbar in the T-Wave cell). Mass Lynx v 4.2 software (Waters, USA) was used to process quantitative data obtained from calibration standards and samples. The experiments were performed in triplicate.

### 2.5 Drought stress application, management, and plant sampling

In the growth chamber experiments, inoculated seeds were sown in 100 mL plastic pots filled with commercial substrate (Turba Plus, Carluccio) with 20 plants per treatment. Each pot was watered to 100% field capacity (FC) using distilled water until seedlings reached the Zadoks stage 13 (Haun stage 2.6), when a moderate drought stress (MS) corresponding to 40% of FC was applied to each treatment. Watering was withheld until the soil FC reached 40%, and drought stress levels were maintained for 7 d by daily weighing of pots and adding distilled water to compensate for water loss.

After 7 days under drought stress, the uppermost, fully expanded leaves from six plants of each treatment were sampled on the 7th day of drought stress. Six leaves from each treatment were used to determine leaf relative water content (RWC). Leaf RWC was determined according to the standard method proposed by Barrs and Weatherley (1962) as RWC = (FW − DW)/(TW − DW), where FW is fresh leaf weight, DW is dry weight, and TW is turgid weight after 24 h floating in distilled water at 4°C in darkness. Four plants from each treatment were used to analyze root and shoot fresh and dry weight. Roots were subsequently scanned to determine total root length (RL), projected area, and the number of forks per root using the WinRHIZO 2007 software (Regent Instruments, Ottawa, Canada).

### 2.6 Miscellaneous methods

Fresh leaf samples were frozen in liquid nitrogen, powdered with liquid nitrogen, and stored at − 80°C for total sugars and proline determinations.

Sugars in frozen leaf samples (100 mg) were extracted using ammoniacal water (pH 8.0) in 100°C bath for 5 min, followed by centrifugation at 10,000×*g* for 10 min after cooling. Total soluble sugar was measured colorimetrically by the anthrone method at 620 nm (Pontis, 2016; Bader et al., 2024). Glucose was used as a standard for total soluble sugar measurements.

Proline in frozen leaf samples (100 mg) was extracted with 3% (w/v) sulfosalicylic acid, and the extract was centrifuged at 15,200*×g* for 10 minutes. A sample of the clarified extract was combined with sulfosalicylic acid, glacial acetic acid, and acid-ninhydrin and incubated for 1 hour at 96°C. The reaction was halted by placing the tubes on ice. Two milliliters of toluene were incorporated into the mixture, stirred for 20 seconds, and allowed to settle for 5 minutes to facilitate phase separation. The absorbance of toluene (upper layer) was quantified at 520 nm, using toluene as the reference standard. The proline content was quantified utilizing a standard curve according to the methodology established by Bates et al. (1973).

### 2.7 Field trials: experimental design

Field trials were conducted in two distinct years, concurrently at three locations without irrigation, under three different agronomic management systems: No-till (NT, site 1, sowing date June 22th, 2022), Agroecological Management (AM, site 2, sowing date June 22th, 2022), and Conventional Management (CM, site 3, sowing date August 1st, 2024). All trials were carried out at the Balcarce Experimental Station of the Instituto Nacional de Tecnología Agropecuaria (INTA) (37°46′ 14″ S; 58°18′ 23″ W; 113 m.a.s.l.) from June 2022 to January 2023 and from August 2024 to January 2025. According to pre-sowing soil analysis, the soil for the three sites was classified *as Typic Argiudoll* (USDA Taxonomy) and fine thermic Petrocalcic Paleudoll (petrocalcic horizon at 140 cm) with a loamy surface texture and 4.39% organic matter (Site 1), 5.34% organic matter (Site 2), and 4.87% organic matter (Site 3). In site 1, the soil contained 18.4 P_2_O_5_ (ppm, 0–20 cm depth), 14.1 N-NO_3_ (ppm, 0–20 cm depth), and 8.5 N-NO_3_ (ppm, 20–50 cm depth). In site 2, organic manure was applied at sowing, and the soil before planting contained 15.1 P_2_O_5_ (ppm, 0–20 cm depth), 22.6 N-NO_3_ (ppm, 0–20 cm depth), and 6.3 N-NO_3_ (ppm, 20–50 cm depth). In site 3, the soil contained 34 P_2_O_5_ (ppm, 0–20 cm depth), 1.36 N-NO_3_ (ppm, 0–20 cm depth), and 1.57 N-NO_3_ (ppm, 20–50 cm depth). For NT (year 2022, site 1), bread wheat was established in the residue of the preceding crop (soybean). For AM and CM (year 2022, site 1 and 2024, site 3), agronomic field operations before sowing include moldboard plowing to a depth of 30 cm, followed by seedbed preparation with a disc harrow. In site 3, summer crop (soybean) residues were incorporated during plowing. In sites 2 and 3, chemical fallow mulching was performed in the fall of both 2022 and 2024 (Paraquat 2,5 L.ha^-1^). Dry soil was fertilized at the planting line with 150 kg.ha^-1^ of diammonium phosphate and with 408 kg.ha^-1^ of nitrogen, as urea distributed in two moments, at tillering and at the beginning of the stem elongation period. Fertilizer timing was managed relative to rainfall forecasts to ensure nutrient availability. Weeds, pests, and fungal diseases were chemically controlled. For 2022 campaign: Herbicide: 09/07/22 Hussar-pluss (240 cm^3^.l^-1^) + Metsulfuron (5 g.l^-1^) + coadyvant. Fungicide: 11/02/22 and 11/10/22 Cripton-Xpro 500 cm^3^.ha^-1^. For 2024 campaign: Herbicide: 10/02/24 Hussar-pluss (240 cm^3^.ha^-1^) + Metsulfuron (5 g.ha^-1^) + coadyvant. Fungicide: 11/11/24 Orquesta Ultra (fluxapyroxad 50 g.l^-1^, epoxiconazole 50 g.l^-1^ and pyraclostrobin 81 g.l^-1^) 1000 cm^3^.ha^-1^.

Each assay consisted of four blocks, each 5 meters long, interspersed by a 2-m path. At sowing, the plots comprised 7 furrows spaced every 20 cm and 6.5 m long (6.5 x 1.4 m). Each plot was sown with a density of 350 plants.m^-1^ (high density). Around the experimental blocks, durum wheat was sown to reduce the edge effect in the trial. Harvest was performed mechanically along the five central rows of each plot (5 x 1 m). Daily meteorological data were obtained throughout the test by the meteorological station located in the experimental field (INTA-Balcarce, **Table 1**; **Supplementary Figures S1, S2**). Phenological stages, biomass, and yield components were determined according to Pask (2012). For each treatment, plant count. m^-1^, the number of tillers per plant, dry weight of aerial part and roots, and radical architecture, were analyzed at tillering. Biomass, harvest index, grain yield (GY), and grain quality were analyzed at harvest. Biomass was determined by sampling 2 linear m of each plot and weighing total dry aerial part. Harvest index was calculated as the percentage of total grain weight of the samples after threshing and biomass. Grain weight (GW) was determined by counting a 1,000-grain sample with an electronic counter, weighing it, and dividing the total weight by 1,000. Grain number per ha was then calculated as the quotient between GY and GW × 1,000. Test weight was determined with a 500 mL-Schopper chondrometer. Based on wheat GY and precipitation, CWP was calculated (kg. m^−3^).

**Table 1:** Monthly cumulative precipitation events during the past decade (historical precipitation) and for the years 2022 and 2024.

Month	Hist. Precip	Precip. 2022	Precip. 2024
January	92,7	67,75	69
February	90,7	84,5	97,3
March	90,0	88,75	94
April	85,8	58	51,2
May	51,9	10,25	7,7
June	55,2	2,5	13,4
July	50,6	48,25	6,6
August	72,5	20	64,4
September	67,4	10,5	32,5
October	67,1	40	13,1
November	90,6	99,5	199
December	71,6	54,5	168,5

### 2.8 Statistical analyses

For all sites, a randomized blocked design (RBD) was made in which the inoculation factor was analyzed at six levels with 4 repeats. The arrangement of the blocks was planned considering the inclination of the lot. For tilling, discrete data Odds Ratio Pairwise Comparison Analysis was used. One-way analysis of variance (ANOVA) followed by *post-hoc* Fisher LSD test or Tukey’s test, were used to detect significant differences (p < 0.05) between treatments. All analyses were performed in GraphPad Prism 7.04 or Python software (Odds Ratio Pairwise Comparison Analysis).

## 3 Results

### 3.1 Co-inoculation of *Azospirillum* with microalgae increases bacterial root colonization of wheat plants

**Figure 1:**
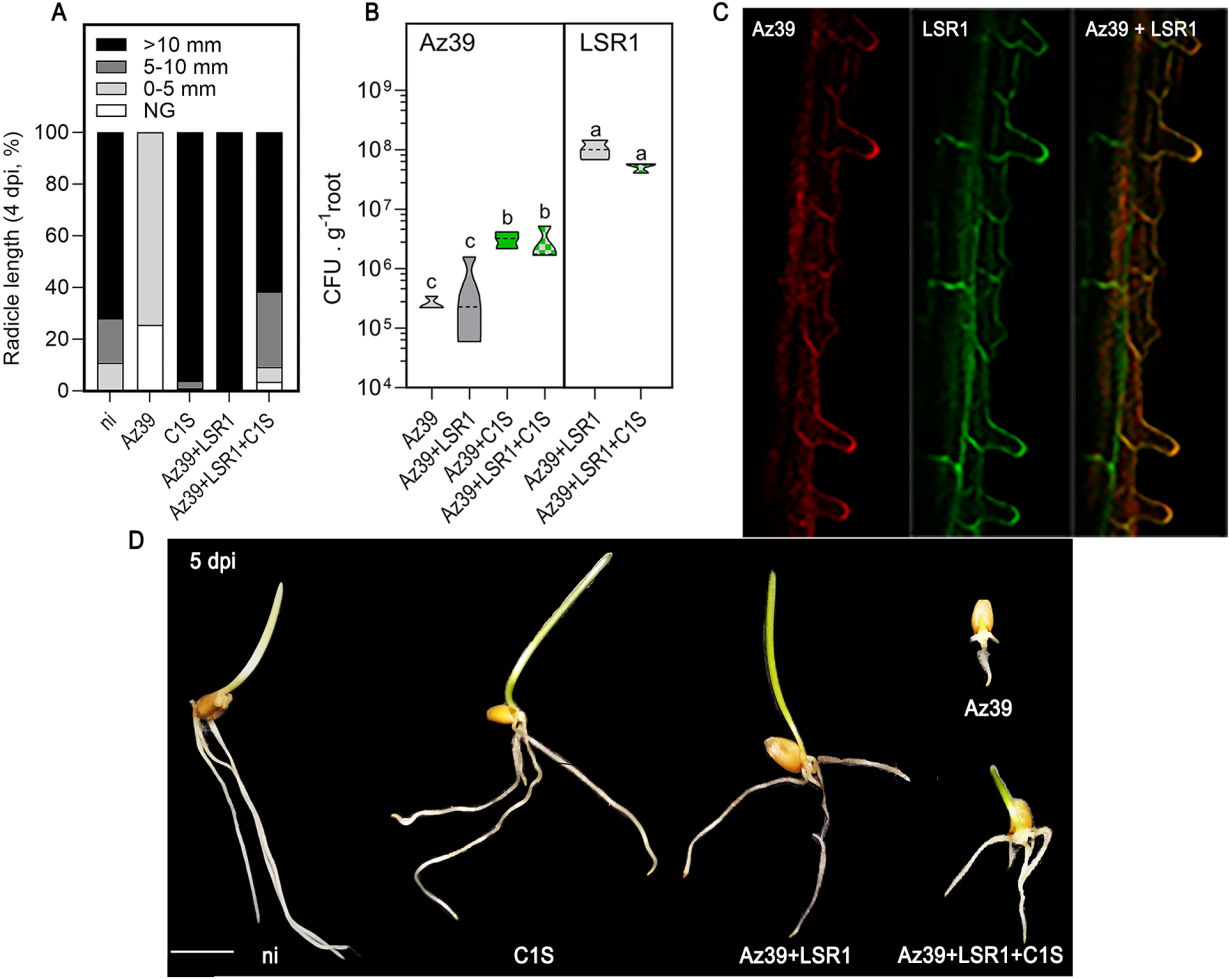
Germination, early root growth, and bacterial root colonization in inoculated seedlings. The percentage of seedlings non-germinated (NG), with 0-5, 5-10, or more than 10 mm radicle length after 4 d post-imbibition (dpi), was compared among non-inoculated (ni) and inoculated seedlings **(A)**. *A*. *argentinense* Az39 (left panel) or *Pseudomonas* sp. LSR1 (right panel) root colonization was measured as CFU. g^-1^ of root fresh weight **(B)**. Bacterial strains distribution on the roots of seedlings was analyzed at 5 dpi by confocal laser scanning microscopy **(C)**. Phenotype comparison of seedlings of the different treatments at 5 dpi **(D)**. Bar scale: 1 cm. Data were statistically analyzed by one-way ANOVA. Different letters on the graphs indicate significant differences according to Tukey's post-test (p ≤ 0.05).

Since the early interaction between plants and microorganisms can significantly influence seedling establishment, germination and post-germinative growth was evaluated upon microalgal co-inoculation with well-established models of PGPB (Az39 and LSR1). The single inoculation with *A. argentinense* Az39 delayed germination and early root elongation. Nonetheless, its co-inoculation with *Pseudomonas* sp. LSR1 not only prevented a delay in germination and growth, but also augmented these processes significantly (**Figures 1A, D**). Likewise, the single inoculation with the microalgae *S. obliquus* C1S stimulated germination and post-germinative development. Conversely, inoculation with the full community (Az39+LSR1+C1S) did not affect the initial seedling growth markedly (**Figures 1A, D**). These results show that each alternative inoculant (single, double, and triple) differentially affects seedling establishment, possibly due to hormone interactions on the seed priming process.

**Figure 2:**
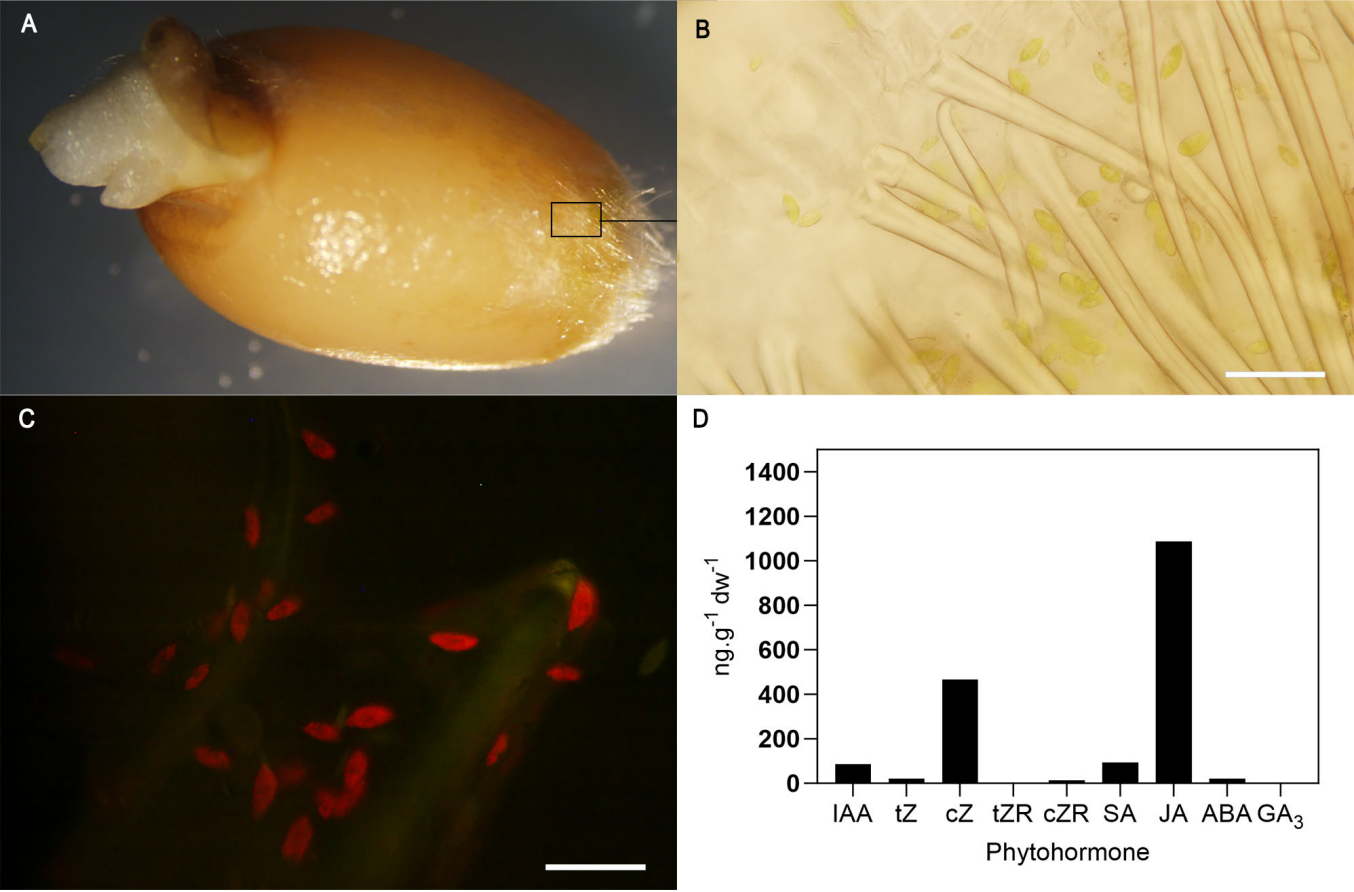
Microalgae localization in germinating seedlings and phytohormone profile. Microalgal localization observed in radicle protruding seeds (1 dpi) with an optical magnifier **(A)**. Transverse sections (1 mm thick) of the trichome region of the seed **(B)** were stained with SYTOX Green, and both chlorophyll autofluorescence and green fluorescence were analyzed by light and fluorescence microscopy **(C)**. Bar scale: 20 μm **(B, C)**. *S. obliquus* C1S phytohormonal profile. Levels of IAA, tZ, cZ, tZR, cZR, SA, JA, ABA and GA_3_ were analyzed by UHPLC-MS/MS **(D)**.

A prerequisite for plant-growth-promoting bacteria (PGPB) to promote plant growth is the establishment of a stable bacterial population on the roots (Okon and Labandera-Gonzalez, 1994; Creus et al., 2004). However, when bacteria are inoculated on dry seeds, their survival and viability often diminish quickly, hindering root colonization (Köhl et al., 2024). To determine whether bacterial performance after seed inoculation can be improved by its co-inoculation with microalgae, PGPB root colonization was evaluated in wheat (*Triticum aestivum*) at 3 d post-germination*. A. argentinense* Az39 root colonization was approximately 10-fold higher when it was co-inoculated with the microalgae (3.19 x 10^6^ CFU.g^-1^), either in the presence or absence of *Pseudomonas* sp. LSR1. Conversely, LSR1 root colonization exhibited consistency, independently of the presence of C1S (**Figure 1B**). As expected, both bacteria were primarily located in the elongation zone, especially on root hairs for Az39, whereas LSR1 exhibited a more uniform distribution (**Figure 1C**). Microalgae localization was also analyzed in imbibed and germinating seeds. As observed in **Figures 2A, B**, microalgae were mostly located within the trichomes of the seed coat during germination, and a release of microalgae into the surrounding medium was also noted. No microalgae were observed on the radicle or the apical axis during the early establishment of seedlings. Microalgae viability was also examined by a dual-fluorescence viability experiment using SYTOX Green along with chlorophyll autofluorescence as a contrast marker, to identify dead and living microalgal cells (Sato et al., 2004). As shown in **Figure 2C**, no SYTOX Green fluorescence was observed, indicating that all seed-attached microalgae were viable. *S. obliquus* phytohormone profile revealed high concentrations of JA and cZ, moderate levels of SA and IAA, and reduced amounts of tZ, cZR, and ABA. GA_3_ and tZR were not detected (**Figure 2D**).

### 3.2 Microalgae inoculation under conventional tillage enhanced wheat tillering and root weight

To analyze inoculated wheat treatments under agronomic scenarios, both NT and conventional tillage were explored. Wheat field experiments were conducted in Balcarce, Buenos Aires, Argentina, in June 2022 with conventional tillage and agroecological management (AM) or NT, and in August 2024 under conventional management (CM). The field trials were conducted using a completely randomized design with four repetitions and six inoculation treatments: Az39, C1S, Az39+C1S, Az39+LSR1, Az39+LSR1+C1S, and a control without inoculation.

**Figure 3:**
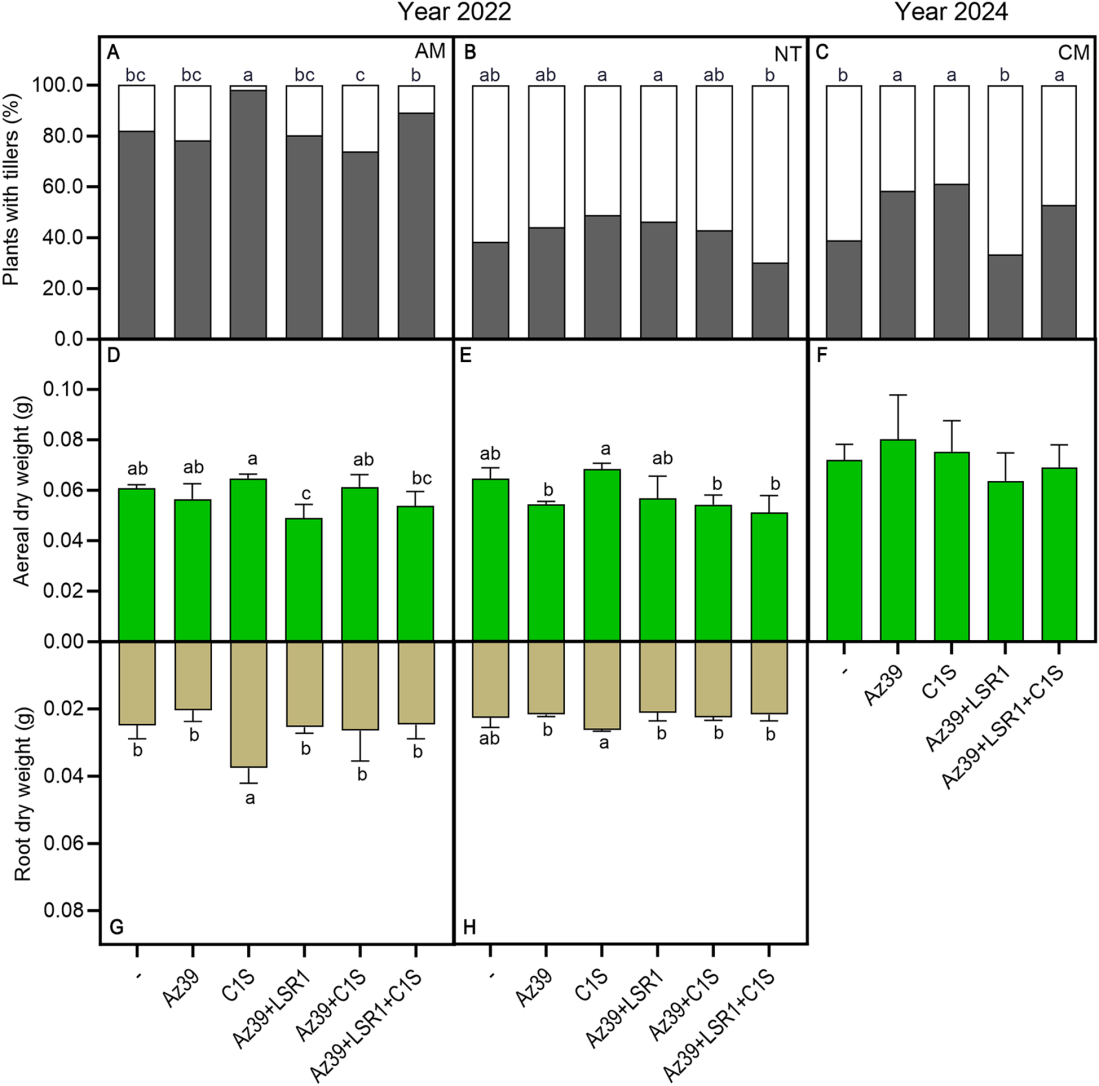
Microalgae and PGPB-inoculated wheat vegetative growth in the field under different management practices. The percentage of plants with at least one tiller (black) or without tillers (white) was compared among treatments by an Odds Ratio (OR) Pairwise Comparison Analysis in AM **(A)**, NT **(B)**, and CM **(C)**. Aerial **(D–F**) and root **(G, H)** dry weights in AM **(D, G)**, NT **(E, H)**, and CM **(F)** were measured at tillering. One-way ANOVA followed by *post-hoc* Fisher LSD test was used to detect differences between means. Different letters on the bars indicate significant differences between treatments (p ≤ 0.05).

Microalgae, Az39, and Az39+LSR1+C1S treatments increased wheat tillering only under conventional tillage (AM and CM; **Figures 3A, C**; NT: **Figure 3B**). No significant differences were found in aerial dry weight between the inoculation treatments and the control group (**Figures 3D–F**). However, the root dry weight of C1S-inoculated seedlings under AM was higher than in the non-inoculated ones (**Figures 3G, H**). Under CM, the roots could not be collected as a whole, rendering them unanalyzable (results not shown).

### 3.3 Microalgae-PGPB inoculation enhances grain wheat productivity in the field

**Figure 4:**
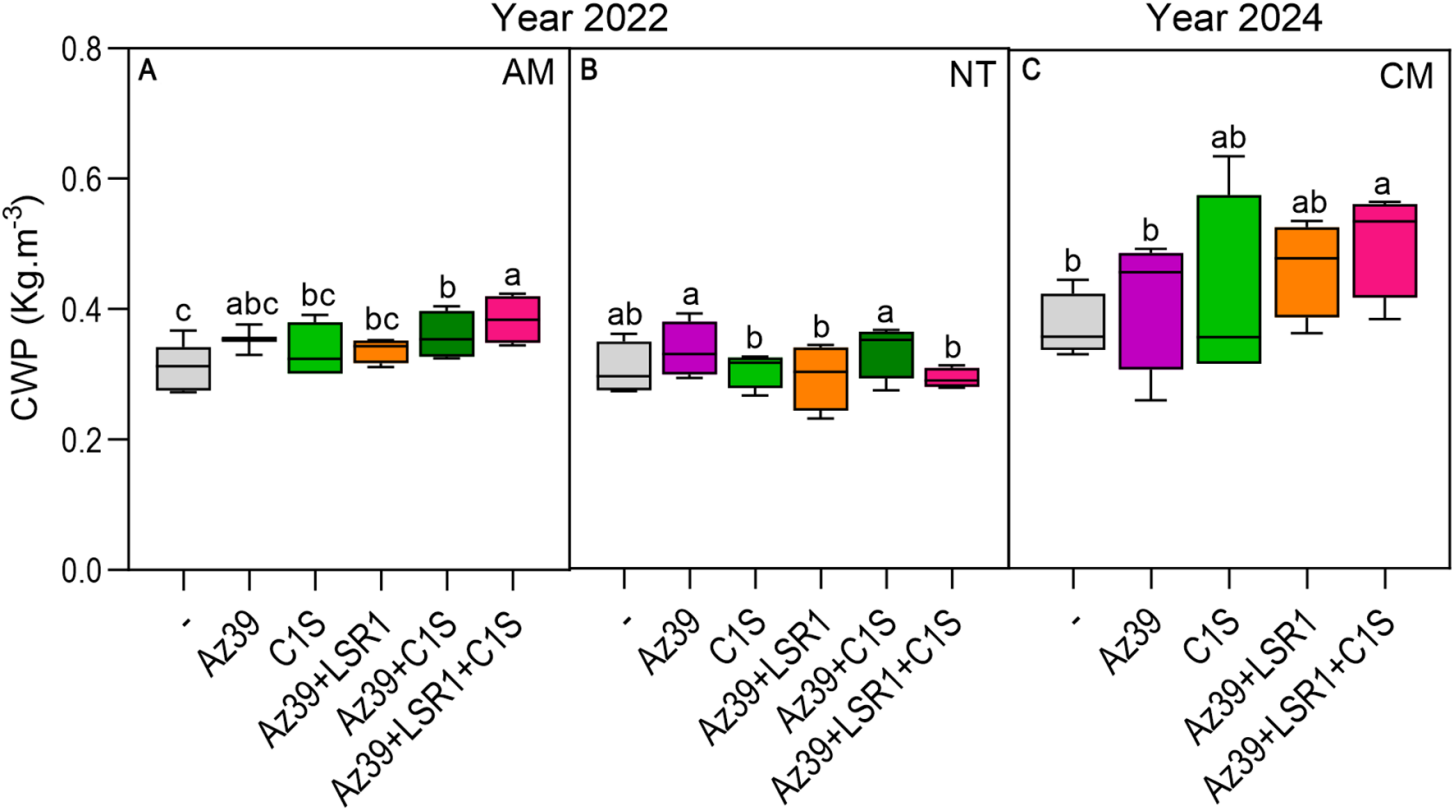
Crop water productivity under different field managements. The CWP of wheat was measured under AM **(A)**, NT **(B)** and CM **(C)**. One-way ANOVA followed by the *post-hoc* Fisher LSD test was used to detect differences between means. Different letters on the bars indicate significant differences between treatments (p ≤ 0.05).

Inoculated plants showed a 36% overall increase in GY by the triple inoculation and a 14% and 26.2% increase in crop water productivity (CWP), only under tillage management (AM and CM, respectively). The positive effect of the triple consortium on yield and CWP was also consistently observed under tilled conditions (AM and CM) but was absent under no-till (NT; **Table 2**, **Figure 4**).

**Table 2:** Effect of inoculation treatments and field managements on the yield components in wheat.

Parameter	Year	Management	Uninoculated	Az39	C1S	Az39+LSR1	Az39+C1S	Az39+LSR1+C1S
Number of spikes (spikes. m^-1^)	2022	AM	338.7 ± 30.7a	321.6 ± 68.6ab	247.5 ± 31.1b	322.5 ± 79.6 ab	292.5 ± 33.4ab	260.0 ± 46.4b
		NT	476.2 ± 115.23a	675.0 ± 115.0a	536.2 ± 53.3a	611.2 ± 56.8a	538.3 ± 39.2a	598.8 ± 30.9a
	2024	CM	303.7 ± 49.9b	336.2 ± 32.6ab	326.3 ± 60.4ab	387.5 ± 55.9ª	nd	390.0 ± 33.0a
Kernels per spike (^-1^)	2022	AM	28.1 ± 2.8b	31.9 ± 3.1ab	41.2 ± 4.6a	29.9 ± 1.4b	36.5 ± 2.2a	40.4 ± 4.8a
		NT	34.6 ± 4.1ab	30.4 ± 3.9b	35.7 ± 2.4a	29.0 ± 4.0b	36.7 ± 5.0ab	30.1 ± 1.5b
	2024	CM	21.7 ± 1.5a	21.8 ± 2.5a	21.9 ± 3.1a	21.7 ± 4.0a	nd	22.6 ± 3.1a
Number of grains (m^2^)	2022	AM	5123 ± 501.5a	5454 ± 402.4a	5338 ± 587.7a	5211 ± 245.0a	5658 ± 382.1a	5740 ± 595.5a
		NT	4884 ± 568.5a	5367 ± 632.0a	5007 ± 357.4a	4668.± 668.6a	5466 ± 637.6a	4583 ± 99.5a
	2024	CM	6529 ± 751.5b	7393± 1458.3b	7227± 2155.3ab	8205 ± 910.1ab	nd	8794 ± 1243.6a
Test weight (Kg. hl^-1^)	2022	AM	75.8 ± 0.26a	76.1 ± 0.3a	75.8 ± 0.4a	76.1 ± 0.3a	75.9 ± 0.2a	75.6 ± 0.2a
		NT	74.3 ± 0.5b	74.5 ± 0.4ab	74.5 ± 0.8a	74.3 ± 0.4b	74.2 ± 0.4b	74.4 ± 0.6ab
	2024	CM	81.4 ± 0.5a	80.9 ± 0.4a	81.2 ± 0.1a	81.2 ± 0.2a	nd	81.3 ± 0.4a
1000-grain weight (g)	2022	AM	36.9 ± 0.6ab	37.3 ± 1.1ab	36.7 ± 0.7b	37.9 ± 0.1a	37.1 ± 1.2ab	38.3 ± 0.9a
		NT	36.7 ± 0.35a	36.7 ± 0.9a	35.8 ± 0.5a	36.9 ± 1.1a	36.0 ± 1.2a	36.4 ± 1.2a
	2024	CM	46.7 ± 0.78a	45.8 ± 1.5a	46.9 ± 0.9a	46.1 ± 1.8a	nd	46.9 ± 1.0a
Grain yield (Kg. ha^-1^)	2022	AM	1927 ± 196.3b	2065 ± 111.5ab	1957 ± 216.0b	1972 ± 396.2b	2100 ± 186.3ab	2244 ± 190.9a
		NT	1791 ± 205.7ab	1964 ± 214.9a	1791 ± 139.1b	1724 ± 258.3b	1964 ± 213.0a	1709 ± 78.1b
	2024	CM	3046. ± 354.7b	3406 ± 753.4b	3402 ± 1069.4ab	3792 ± 524.2ab	nd	4130 ± 586.6a
Harvest index (%)	2022	AM	36.9 ± 1.9a	35.9 ± 0.4a	38.2 ± 1.9a	36.7 ± 1.2a	37.8 ± 2.4a	38.0 ± 1.8a
		NT	31.2 ± 0.5a	25.2 ± 8.1a	27.0 ± 8.3a	24.9 ± 7.9a	33.2 ± 1.1a	30.2 ± 3.1a
	2024	CM	45.6 ± 0.9b	47.1 ± 2.2b	44.4 ± 3.2b	48.3 ± 1.5a	nd	46.9 ± 2.2b

Data are presented as mean ± SD. Different letters in each row indicate significant differences among treatments within each management system (AM, NT, CM) for a given year and parameter at p < 0.05. nd: not determined.

**Table 2** shows that the yield components that contribute to the increase in productivity observed with Az39+LSR1+C1S under AM and CM differed. Under AM, the number of kernels per spike was 46,6%, 29.9% and 43.7% higher in C1S, C1S+Az39, and Az39+LSR1+C1S, respectively, compared to the non-inoculated plants. A marginal enhancement of 2.7% and 3.8% in thousand-grain weight occurred in the Az39+LSR1 and Az39+LSR1+C1S treatments, respectively; however, this variation was not statistically significant.

On the other hand, under CM, the number of kernels per spike did not reveal any differences between the inoculated and control plants. But, interestingly, the number of spikes.m^-2^ in Az39+LSR1, and Az39+LSR1+C1S treated plants were 27.6% and 28.4% higher, respectively. Consequently, the number of grains.m^-2^ were 25.7% and 34.7% higher for Az39+LSR1- and Az39+LSR1+C1S-treated plants, respectively (**Table 2**).

Historical records from Meteorological Services in Argentina indicated that during 2022 (Buss et al., 2023) and 2024 (SMN, 2024), the crop season was under the influence of ´La Niña´ ENSO stage, resulting in significantly reduced precipitation recorded at the INTA weather station during most of the crop cycle (**Table 1**; **Supplementary Figure S1**).

In both 2022 and 2024 crop seasons, water scarcity was especially severe throughout the plant’s tillering stage (Zadoks stage 2, mid-September, **Table 1**; **Supplementary Figure S3**). The soil water balance model, based on soil, climate, and satellite data, estimated available water content to be below 10% and between 10% and 20% at the end of September 2022 and 2024, respectively (**Supplementary Figure S3**). Consequently, to thoroughly examine the effect of inoculation on drought resilience, wheat plants response under induced drought stress in chamber experiments were studied.

### 3.4 Microalgae and microalgae-PGPB inoculation confer drought tolerance to wheat seedlings

**Figure 5:**
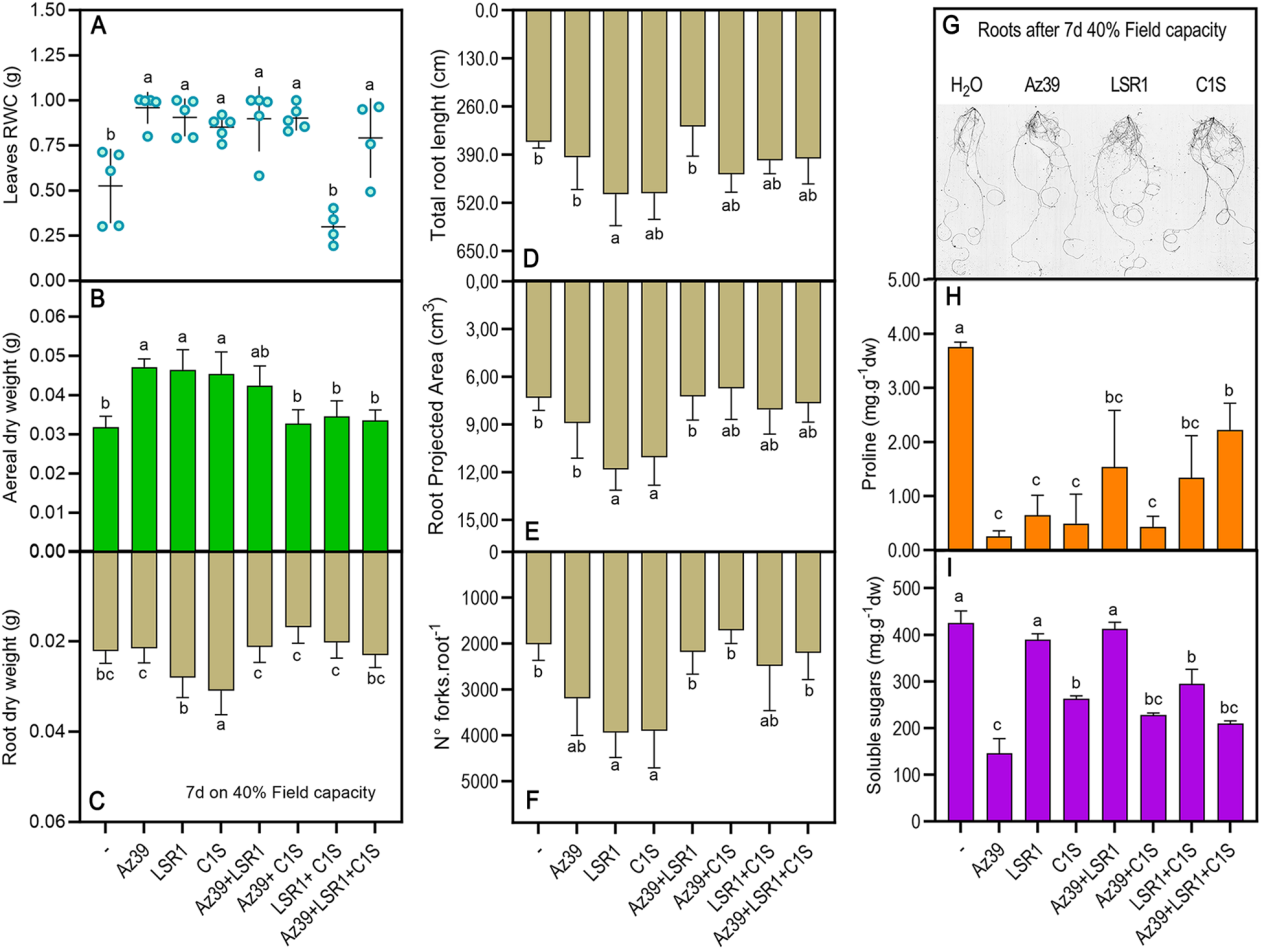
Drought stress response of inoculated wheat seedlings. Analysis of RWC **(A)**, aerial **(B)** and root **(C)** dry weight, total root length **(D)**, projected area **(E)**, and number of forks **(F)**. Representative scan images of treated roots are also shown **(G)**. Proline **(H)** and soluble sugar content **(I)** in the aerial portion were measured. Data were statistically analyzed by one-way ANOVA. Different letters on the histograms indicate significant differences according to Tukey’s post-test (p ≤ 0.05).

To assess if seed inoculation could promote drought stress resilience, suspensions with Az39, LSR1, C1S, Az39+LSR1, Az39+C1S, LSR1+C1S, or Az39+LSR1+C1S were inoculated on dry seeds, which were sown in pots watered at field capacity until seedlings reached the Zadoks stage 2. At this point, seedlings were maintained under moderate drought stress condition (MS) in a growth chamber for 7 d. The relative water content (RWC) of wheat leaves of Az39, LSR1, C1S, Az39+LSR1, and Az39+LSR1+C1S treatments was higher than that of the untreated control (**Figure 5A**). Shoot aerial dry weight was 55% higher in plants treated with Az39, LSR1, or microalgae C1S individually, when compared to the non-inoculated plants (**Figure 5B**). Furthermore, LSR1 and C1S single-inoculation treatments differentially modified the root architecture upon drought stress, increasing the total root length, projected area, and branching (**Figures 5D–G**). However, in accordance with field experiments, only microalgae-inoculated seedlings significantly increased root dry weight by a mean of 50% (**Figure 5C**).

As expected, the osmoprotectants proline and soluble sugars (SS) accumulated in non-inoculated drought-stressed seedlings (**Figure 5H**), indicating a clear drought stress response. In contrast, all inoculation treatments prevented proline accumulation (**Figure 5H**). Conversely, sugar content was diminished in all the treatments containing microalgae and in Az39 single inoculation (**Figure 5I**).

## 4 Discussion

Our findings revealed that co-inoculation with *Pseudomonas* strain LSR1 or microalgae *Scenedesmus obliquus* C1S increases *Azospirillum argentinensis* Az39 root colonization by tenfold (**Figure 1**). Furthermore, we show that the single inoculation with *Azospirillum*, presumably due to its ability to produce abscisic acid (ABA), delays germination and early root elongation (Cassán et al., 2020). Nonetheless, co-inoculation with LSR1 and with LSR1 and C1S counteracts this inhibition by Aza39, presumably through an opposing phytohormonal effect (Wang et al., 2011; Maroniche et al., 2016). Notably, inoculation with microalgae also stimulates germination and post-germinative growth. This seed priming phenotype induced by microalgae inoculation could also be explained by its phytohormone profile, mainly enriched in JA and cis-Zeatin (**Figure 2**). It has been reported that seed treatment with elicitors like JA or cytokinin can enhance seed germination rate, particularly under stressful conditions (Iqbal et al., 2006). ABA and cytokinin exert antagonistic effects on the regulation of plant growth. Increased cytokinin levels can induce the downregulation of ABI5 expression via transcriptional regulators, and the ABA-inhibited seed germination is alleviated (Wang et al., 2011). These findings provide a new perspective on the properties of eukaryotic microalgae, specifically regarding their hormonal release on seed surfaces, the priming of seedlings, and the introduction of distinctive traits into microalgal-bacterial synthetic consortia formulations, enhancing bacterial root colonization abilities.

Field experiments revealed that microalgal inoculation increased wheat tillering and root dry weight. Moreover, a 36% increase in GY and a 26.2% increase in CWP were observed in response to the inoculation with microalgae-PGPB triple consortia. Interestingly, inoculation efficiency is significantly affected by tillage practices before sowing, since GY and CWP remained unchanged under NT for all treatments (**Figure 4**, **Table 2**). According to several reports, tillage emerged as the main factor shaping the microbial community composition in the rhizosphere (Romano et al., 2023; Symanczik et al., 2025), and NT shows an increased soil extracellular enzyme activity (Li et al., 2024). Consequently, the absence of crop yield increase in response to inoculation under NT could be attributable to a more robust and competitive rhizospheric microbiome that outcompetes the introduced consortium.

On the other hand, under NT, the top soil exhibits more compaction relative to conventional tillage (Lipiec et al., 1995). Physical alterations in soil resulting from no-till practices might adversely impact the development of primary root axes, especially during the early phases of plant development (Ferreira et al., 2021). Heightened resistance resulted in an exponential reduction in root length (Martino and Shaykewich, 1994). Since root exudates play a crucial role in shaping the soil microbiome, to maximize the functionality of beneficial bacteria, it is essential to apply them to crops in ways that align with environmental conditions that also favor root development. These findings highlight the necessity of evaluating microbial inocula while considering soil management into account in order to maximize the growth-promoting effects in productive scenarios.

The seasons in which we conducted this research in the field represent an interesting case study representing possible future conditions related to climate change. Drought conditions on the vegetative stages of wheat growth (June to mid-September, **Table 1**, **Supplementary Figures S1, S3**) impacted throughout the South American region in 2022–2023- and -to a lesser extent- in 2024-2025, both under the influence of the ENSO stage ´La Niña´. Between 2019 and 2024, persistent La Niña conditions in Argentina correlated with an exceptional drought, exacerbated by several hot waves throughout the entire country (Lopez-Ramirez et al., 2024). In this context, field experiments suggested that wheat plants’ resilience to water scarcity under field conditions was enhanced by the Az39+LSR1+C1S synthetic community inoculation. Chamber trials under drought stress exhibit a 55% enhancement in aerial dry weight with the sole inoculum of Az39, LSR1, and microalgae C1S. Additionally, as observed under field trials, seedlings inoculated with microalgae showed an average increase of 50% in root dry weight and had a greater effect on total length, projected area, and higher root branching than non-inoculated plants.

Osmoprotectants shelter organisms from stress by acting as osmolytes, and the most important osmolytes found in plants include sugar alcohols, soluble sugars, polyols, proline, and betaine (Mohammadi Alagoz et al., 2023). As proline is a thoroughly studied osmoprotectant, its determination represents a very valuable analytical tool to probe the physiological status of plants regarding drought conditions (Mu et al., 2021). In this context, sugar content in drought-stressed plants was diminished in all the treatments containing microalgae. We interpret this result as follows: being sugars osmoprotectants in response to stress, stress-alleviated plants after inoculation might accumulate lower levels of sugars. The effect might be similar to that of proline accumulation. Considering that all these treatments showed enhanced biomass, sugars may have been used as a carbon source for growth rather of being retained as osmoprotectants. Conversely, proline levels appear to more closely reflect physiological stress than sugar levels, as plants treated with Az39, LSR1, and C1S have the lowest proline levels, a high relative water content in leaves, and also significantly greater aerial dry weight, indicating an improved water status of the plants. These findings align with a recent study indicating that *Azospirillum* inoculation reduces proline accumulation via delta-1-pyrroline-5-carboxylate synthase (P5CS) in wheat under water-deficit stress (Karimi et al., 2025). The results indicate that inoculated plants have a mitigated stress response. Drought stress markedly decreases the cytokinin/ABA ratio in shoots, whereas inoculation with the cytokinin-producing PGPB *Bacillus subtilis* increases this ratio by about fivefold, hence enhancing drought stress tolerance (Vurukonda et al., 2016). The present study demonstrates that CK-producing microalgae enhance plant tolerance to drought stress, maybe through the synthesis of this phytohormone. Furthermore, the augmented root projected area noted in Az39, LSR1, and C1S-treated plants may further boost the water potential of these plants.

Overall, the strongest response, in terms of growth enhancement and reduction in stress response under induced drought conditions, was observed with the single inoculation of the microalgae. The detection of microalgae on plant tissues only throughout the initial days post-root emergence (**Figure 2**) suggests that the phenotypic adjustments and the mitigation of drought stress resulting from microalgae inoculation, at least in growth chamber studies, can be attributed to early seed priming, supported by the increased concentrations of cytokinin and jasmonic acid in the phytohormone profile of the microalgae, in addition to other potential biostimulant metabolites secreted on the seed surface.

Alternatively, microalgae released on the soil can exert other beneficial effects on the plant, either by hormonal release, and/or by altering the rhizosphere microbiome.

## 5 Conclusion

The results presented herein demonstrate a promising prospect for the development of a novel eukaryotic microalgae-PGPB synthetic consortia inoculant that boosts root colonization by PGPBs and enhances wheat crop water productivity under challenging field conditions, acting as a complementary strategy for climate change adaptation to ensure food security. To the best of our knowledge, this study is the first to demonstrate a direct inoculation of eukaryotic microalgae on wheat seeds with growth-promoting properties for plants under drought stress. It is noteworthy that under both growth chamber and field conditions, these microalgae are fully compatible with rhizosphere bacteria, even amplifying their growth-promoting activities. The fact that drought protection under drought stress could be provided by eukaryotic microalgae seed inoculation raises interesting biotechnological ramifications to explore. Further research into the physiological mechanisms behind this stress tolerance prompted by this treatment will present new insights into the use of eukaryotic microalgae in agronomic scenarios.

## Data Availability

The original contributions presented in the study are included in the article/**Supplementary Material**. Further inquiries can be directed to the corresponding author.
