# Supplementary figures and images for: Eukaryotic microalgae-bacteria synthetic consortia boost crop productivity and drought tolerance in bread wheat (Triticum aestivum)
Source: Front Plant Sci. 2026 Jan 7;16:1726084. doi: 10.3389/fpls.2025.1726084 (PMC12819807; doi:10.3389/fpls.2025.1726084)

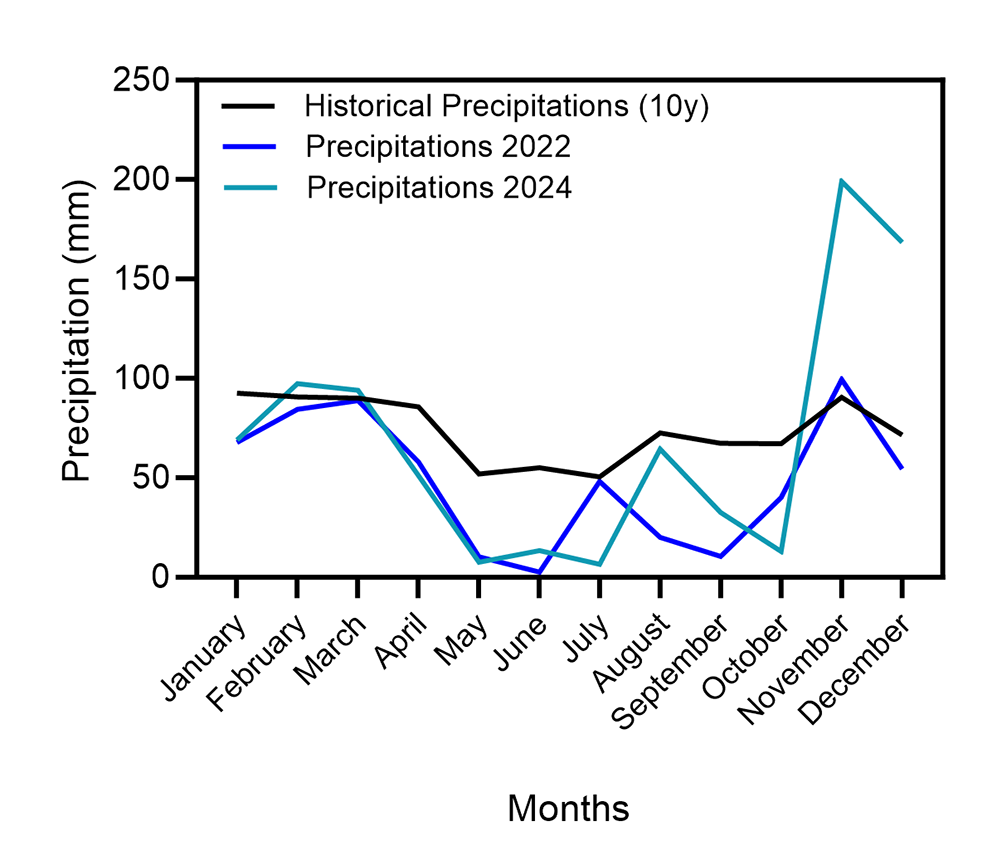

Supplement: Supplementary file 1 [file Image1.tif]

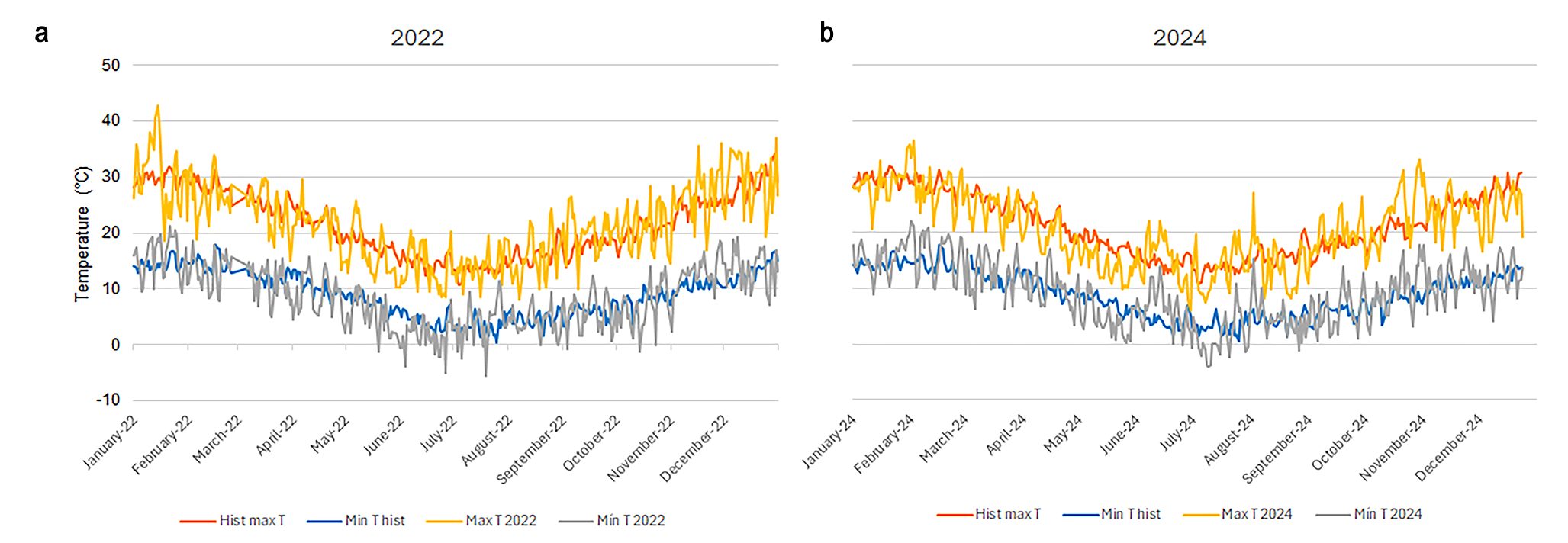

Supplement: Supplementary file 2 [file Image2.tif]

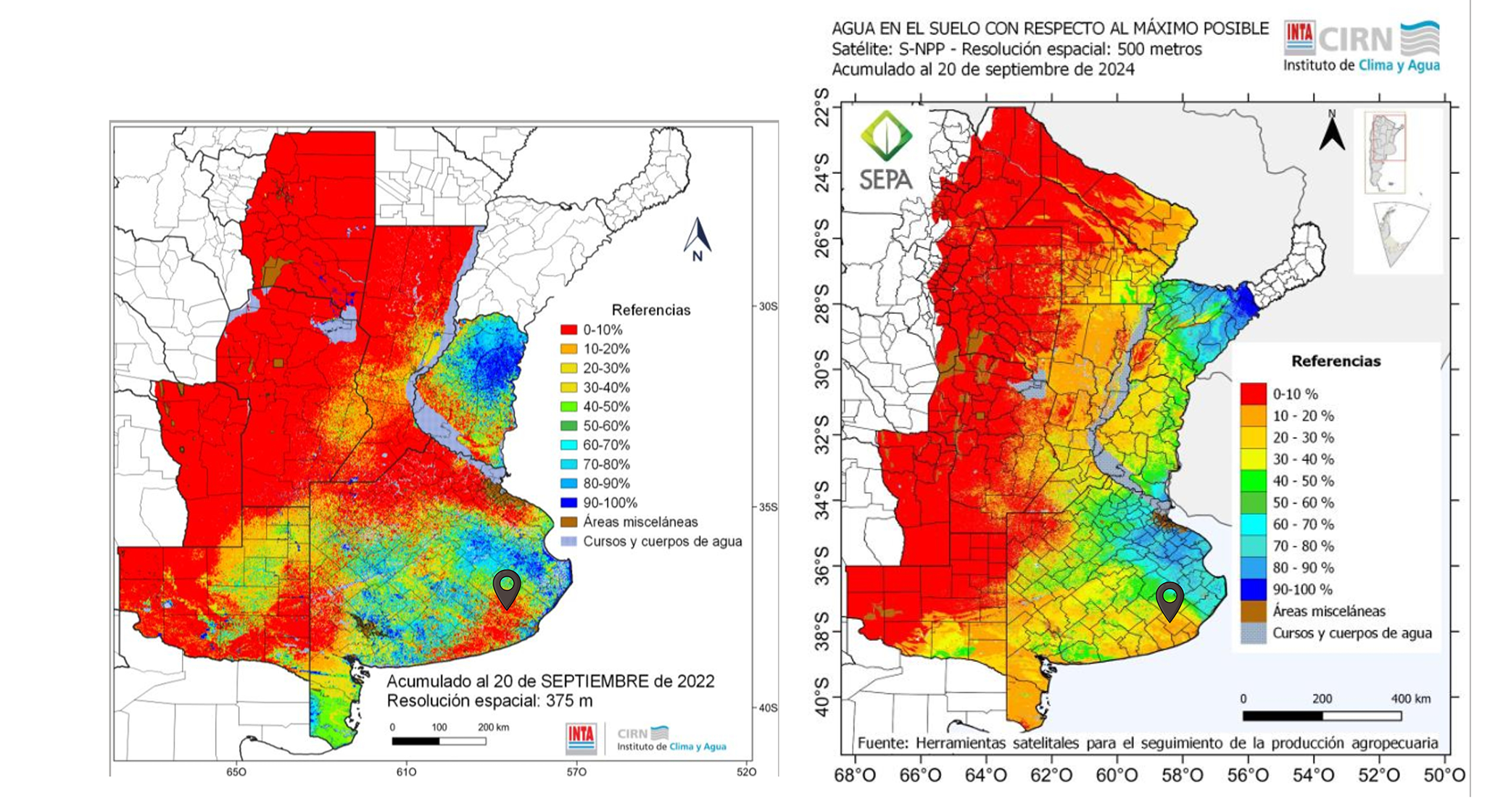

Supplement: Supplementary file 3 [file Image3.tif]
